# Uptake of cervical cancer screening and associated factors among HIV positive women attending adult art clinic at public hospitals in Addis Ababa, Ethiopia, 2022

**DOI:** 10.1186/s12905-024-03169-x

**Published:** 2024-06-28

**Authors:** Fenta Reta Zegeye, Temesgen Dessalegne Legasu, Fekade Demeke Bayou, Mohammed Ahmed Ali

**Affiliations:** 1 Department of General public Health, Addis Ababa Medical and Business College, Addis Ababa, Ethiopia; 2 Department of Midwifery, College of Medicine and Health Sciences, Jigjiga University, Jigjiga, Ethiopia; 3 Department of Epidemiology, College of Medicine and Health Sciences, Jigjiga University, Jigjiga, Ethiopia

**Keywords:** Cervical cancer, Cervical cancer screening, And human immunodeficiency virus-positive women

## Abstract

**Background:**

Cervical cancer is the second most common malignancy in Ethiopia and first in some African countries. It is six times more likely to occur in positive cases of the human immunodeficiency virus than in the general population. If diagnosed and treated early enough, cervical cancer is both treatable and preventable. However, among Ethiopian women who test positive for HIV, the uptake of cervical cancer screening is low. Its determinant factors were not well studied in the study area. Hence, this study was aimed at filling this information gap.

**Objective:**

To assess uptake of cervical cancer screening services and associated factors among HIV-positive women attending an adult antiretroviral treatment clinic at public hospitals in Addis Ababa, Ethiopia, June 1–30, 2022.

**Methods:**

A cross-sectional investigation was carried out in a hospital. 407 participants in all were chosen using the systematic sampling technique. A pretested interviewer-administered questionnaire was used to collect the data from respondents. The data were entered into Epi data version 4.6 and exported to SPSS version 25 for analysis. Bivariable and multivariable logistic regression analysis was employed. Adjusted odds ratio with its 95% confidence interval and *p* value < 0.05 were used to estimate the strength and significance of the association.

**Result:**

Among a total of 407 respondents, 238 (58.5%), 95% CI (53.5–63.3), HIV-positive women were screened at least once in the last five years. In multivariable analysis, age > 45 years old (AOR = 0.18, 95% CI: 0.053–0.644), number of children (3 children) (AOR = 0.19, 95% CI:0.036-0.979), perception of being susceptible to cervical cancer (AOR = 6.39, 95% CI: 1.79–22.74), knowledge of cervical cancer and its screening (AOR = 19.34, 95% CI: 7.87–47.75), and positive attitude towards cervical cancer screening (AOR = 8.06, 95% CI:3.62–17.91) were significantly associated factors with the uptake of cervical cancer screening.

**Conclusion and recommendation:**

in this study, Age > 45 years, having less than three children, feeling susceptible, knowing about cervical cancer and screening, and having a positive attitude toward cervical cancer screening were significant factor of uptake of cervical cancer screening service. There is a need to strengthen the policy and health education on safe sexual practices and healthy lifestyles through information dissemination and communication to scale up screening service utilization.

## Introduction

Cervical cancer is a cancer of the female reproductive organ. There are two main types of cervical cancer: squamous cell carcinoma and adenocarcinoma, which account for 70% and 25% of cases occurring in the world, respectively. It is caused by highly persistent oncogenic human papilloma virus (hrHPV) infection, particularly human papilloma virus (HPV) types 16 and 18, which are the major cause of 70% of cervical cancer in the world [1].

Human papillomavirus is a sexually transmitted virus that is cleared spontaneously, but in HIV-positive women, it is more likely to persist and progress to invasive cervical cancer due to their immune suppression. Cervical cancer (CC) incidence is six-fold greater among women with HIV infection than in the general population [2]. Cervical cancer is the fourth most common cancer in women worldwide and the second most common and leading cause of cancer deaths in Africa in 2018. With regional variation, sub-Saharan Africa has the highest incidence in the world [3, 4].

The incidence, mortality, and prevalence of CC in Sub-Saharan Africa were 25.2%, 23.2%, and 27.6%, respectively. The incidence, mortality, and magnitude of CC in Ethiopia were 17.3%, 16.5%, and 18.2%, respectively [1–3].

There are various common risk factors that increase the probabilities of getting cervical cancer, such as having multiple sexual partners, starting sexual intercourse early, having low immunity, taking oral contraceptives for a long time (more than 5 years), having an increased number of children (three and more), poor genital hygiene, tobacco use, and having a sexually transmitted disease [5, 6]. It takes 10–20 years to transform from HPV infection to invasive cervical cancer. The premalignant conditions are called cervical intraepithelial Neoplasia (CIN). It is categorized or graded as CIN 1 (mild dysplasia), CIN 2 (moderate dysplasia), and CIN 3 (severe dysplasia). Moderate and severe dysplasia (CIN 2/3) is a high-grade cervical cancer precursor and needs to be treated. Treatment is safe and effective with ablative or excisional techniques [7]. The fact that patients in the precancerous stage do not show a clear symptom until they present at an advanced stage A cervical cancer patient usually develops the following symptoms: abdominal or pelvic pain, foul-discharge vaginal bleeding, heavy or prolonged menstrual periods, abnormal vaginal bleeding (between menstrual periods, after coitus, and menopause), and pain during sexual intercourse [6, 8].

Cervical cancer screening (CCS) is the secondary prevention method of cervical cancer by early detection of precancerous lesions and treatment, which significantly reduces incidence and mortality if a woman is screened once in her lifetime. It is a simple and cost-effective method in addition to protecting their health [9]. The long asymptomatic phase and slow disease progression from persistent infection with oncogenic HPV to invasive carcinoma make cervical cancer a highly preventable cancer through early detection of the precancerous lesion and HPV immunization [10]. The recurrence of cervical cancer after treatment for dysplasia is lower than 1%, and mortality is less than 0.5% [11].There are some screening methods available, including cytological-based tests (Pap smear/glass slide cytology and liquid-based cytology), HPV DNA testing, and visual inspection tests (with acetic acid (VIA) and with Lugol’s iodine (VILI)) [12].

The World Health Organization (WHO) recommends a single visit and treatment approach for women living in resource-limited countries using visual inspection and cryotherapy as primary screening and treatment options, respectively, with regular screening every three years starting at age 25 for HIV positive women and 30 years for the general population [13, 14]. Ethiopian guidelines recommend women start screening at the age of 30–49 years for the general population, and since HIV is diagnosed, regardless of age, once sexually exposed, with an interval of five years irrespective of HIV status [15]. Because of the aggressive nature of the disease in HIV-positive women starting screening since HIV is diagnosed is recommended [7].

The uptake of cervical cancer screening in low and middle-income countries is low when compared to high-income countries due to various personal factors, including lack of general knowledge about cervical cancer and screening, social factors, undeveloped health facilities, and inadequacies in professional education [16].

Cervical cancer is the fourth most common cancer among women globally, with an estimated 750,000 new cases and 311,000 deaths annually in 2018. It affects people worldwide, but the age-standardized incidence rate is high in low and middle-income countries compared with developed countries, where 75/100,000 and 10/100,000 women are affected, respectively. Among those who died worldwide in 2018, nearly 90% occurred in low- and middle-income countries [17]. According to the Globocan 2020 report, 604,000 new cases and 342,000 deaths were estimated worldwide in 2020, with rates remain disproportionately high in LMICs and developed countries (18.8 vs. 11.3 per 100,000 for incidence; 12.4 vs. 5.2 per 100,000 for mortality) [18].

Cervical cancer is the second most common cancer, accounting for 22% of all female cancers and 12% of all newly diagnosed cancers every year, and is the leading cause of cancer death in African women [19]. In sub-Saharan Africa, 21.7% of cancer-related deaths were due to cervical cancer in 2018 [20]. In 2018, the East African region had the highest incidence of cervical cancer, with approximately 52,633 new cases and 37,017 deaths. In 2020, East Africa will also remain a highly affected region with an age-standardized 40.1%/100,000 incidence and 28.6%/100,00 mortality compared with Western Asia’s 4.1%/100.000 incidence and 2.3% mortality [18, 21].

In Ethiopia, cervical cancer is the second most common female cancer, with an estimated 6294 new cases and 4884 deaths, and an age-standardized incidence rate of 18.9 per 100,000 women in 2018 [22]. Its prevalence is high when it comes to HIV-positive women compared to HIV-negative women. Women living with HIV have a six-fold higher risk of developing cervical cancer and a ten-fold earlier risk of developing invasive CC compared to their counterparts because of the high persistence of high-risk human papillomavirus infection [17, 23, 24].

Evidence showed in 2018 that HIV-positive women are more likely to be affected than HIV-negative women because HIV-positive women had higher HPV acquisition and lower HPV clearance than HIV-negative women [22, 25]. The Ethiopian Demographic Health Survey (EDHS) 2016, reports a high prevalence of HIV (3.4%) found in Addis Ababa next to Gambella, and women (1.2%) have two times more prevalence than men (0.6%) [26]. Fortunately, cervical cancer is preventable and treatable if detected at an early stage.

A study reported that the average survival was 81.04% and 67.94% for cervical cancer diagnosed at early stages I and II, respectively, and 23.33% and 20.03% for cases diagnosed at advanced stages III and IV, respectively [26]. But it is a tragedy, studies done in Black Lion Hospital, Addis Ababa, show that about (56.8 − 80%) of cervical cancer patients present at an advanced stage, which very little can be done to treat. Among those late-stage patients, 42.5% came from Addis Ababa, and nearly 62% of them were HIV-positive patients [27–29].

By considering those challenges of cervical cancer, WHO in 2020 reported new recommendation guidelines and global strategies to eliminate the burdens of cervical cancer by 2030 using 90-70-90 targets, Among those targets is by 2030, 90% women should be HPV vaccinated, 70% of women should be screened using the high-performance test at least two times in their lifetime, and 90% should be treated in all countries [17]. Similarly, the five-year National Cancer Control Plan of Ethiopia 2016–2020 set a target to achieve at least 80% screening coverage using VIA as a primary screening method among the target population of women. To achieve this plan by 2020, start providing service without charge in all government health facilities in 2016 [21].

Despite this high vulnerability of low and middle-income (LMIC) countries, their cervical cancer screening uptake is only 44%, with the lowest prevalence among women in sub-Saharan Africa ( country level median 16.9%; range 0.9 − 50.8%) compared with > 60% in high-income countries [18]. High-income countries (HIC) have been able to prevent 80% of cervical cancer by improving cervical cancer screening since the middle of the 19th century [4]. A study reported that Sub-Saharan African women’s major factors that decreased uptake of cervical cancer screening were: fear of the pain of the procedure and positive test outcomes, low level of awareness or knowledge about the case and screening procedure, low-risk perception, cultural and religious factors, lack of spousal support, a possible violation of privacy, waiting time, inaccessibility of the test, they do not know where to get the service, health care providers failure to encourage women to screen [30, 31]. In Ethiopia, the study revealed the uptake of cervical cancer screening for HIV positive women is low (7.8-40.1%) [32]. Despite low cervical cancer screening service utilization in the country, evidence about the factors associated with cervical cancer screening service utilization in Ethiopia, particularly in the study setting, was scarce. Therefore, this study identified the factors influencing cervical cancer screening service utilization among HIV- positive women attending adult art clinics at public hospitals in Addis Ababa, Ethiopia.

## Method and materials

### Study area and setting

The study was conducted in Addis Ababa, Ethiopia. Addis Ababa is the capital city of Ethiopia and the headquarters of the African Union. Administratively, it has 11 sub-cities [33]. In Addis Ababa, 12 public hospitals provide cervical cancer screening services integrated with HIV care using VIA as a primary screening service [34]. Among these four hospitals were selected randomly using lottery methods. Based on the data from their registration logbooks, the following selected hospitals, Gandi Memorial Hospital, Menlik II Comprehensive and Specialized Hospital, Yekatit 12 Hospital Medical College, and RasDesta Memorial Hospital provide ART follow-up services on average every month for 561, 523, 694, and 496 HIV positive women, respectively. A hospital-based cross-sectional study was conducted from June 1–30, 2022.

### Source and study population

All HIV-positive women attending adult ART clinics at the public hospitals in Addis Ababa were a source population. All HIV-positive women attending adult ART clinics at the selected public hospitals in Addis Ababa during the study period were part of study population.

### Eligibility criteria

All HIV-positive women who were volunteers to participate in and attend adult ART clinics in Addis Ababa public hospital during the study period were included in this study.

### Sample size determination

The sample size was determined using the single population proportion formula by considering a 95% confidence interval, a 5% margin of error, and the prevalence of uptake of cervical cancer screening from the previous study in Hawassa (*p* = 40.1%) which provided the largest sample size [35].

Single population formula, *n* = (Za/_2_)^2^pq/d^2^=(1.96)^2^(0.41) (0.59)/(0.05)^2^=372, by considering the non-response of rate 10%.

The final sample size was 409. The required samples size for each hospital was calculated as a proportional allocation to the average monthly client flow. The average monthly client flow was estimated by using the last six months’ client flow from the registration book.

### Sampling techniques

The study was conducted in four randomly selected public hospitals out of a total of 12 public hospitals in Addis Ababa. The selected hospitals were Menlik II comprehensive and specialized hospital, Yekatit 12 referral hospital, RasDesta Damtew memorial hospital, and Gandi memorial hospital. The required number of participants in each hospital was selected by using systematic sampling based on their arrival orders to get the service. The first participant on each day was selected by using a lottery method, and the next participant was included after every fifth interval.

### Study variable

#### Dependent variable

Uptake of cervical cancer screening at least once in the last five years (yes/no).

#### Independent variables

Included socio-demographic characteristics, reproductive health and behavioral factors, awareness and source of information, clinical factors, comprehensive knowledge on CC and CCS, attitude toward CCS, and barriers to CCS.

### Operational definition

#### Uptake of cervical cancer screening

HIV positive women who have been screened at least once in the last five years [35]

#### Awareness of CC and CCS

participants who have ever heard about CC and CCS [36].

#### Good knowledge

The study participants were asked 26 knowledge assessing questions. Each question has “Yes” or “No” options. One point was given for all the correct answers, and zero point for incorrect answers. Then, based on the summative score, the mean score of each individual participant was calculated. Thus, participants who scored mean and above value were considered as had good knowledge [37].

#### Poor knowledge

The study participants were asked 26 knowledge assessing questions. Each question has “Yes” or “No” options. One point was given for all the correct answers, and zero point for incorrect answers. Then, based on the summative score, the mean score of each individual participant was calculated. Thus, participants with a score of below the mean value were considered to have poor knowledge [37].

#### Positive attitude towards cervical cancer screening

Women’s attitude towards cervical cancer screening utilization was measured using eight attitude assessing questions. Each question has five points Likert scale (1 = strongly disagree, 2 = disagree, 3 = neutral, 4 = agree, 5 = strongly agree). Women who scored mean and above value were considered to have a positive attitude and those who scored less than the mean value were considered a negative attitude [37].

#### Drug abuse

participants who currently smoke, alcohol drinks (four or more alcohols per day), and use any drug (injectable or non-injectable) that they do not use for medical purposes [38].

### Data collection tools and procedures

The questionnaire was adapted from previous literature and primarily prepared in English [34, 39]. Then translated to Amharic and back to English to ensure that the translated version gives the proper meaning. An Amharic version questionnaire was used for data collection. The final questionnaire includes socio-demographic, reproductive health, and behavioral factors, awareness and source of information, knowledge of CC and CCS, and attitude toward CCS, clinical factors, and barriers to cervical cancer screening.

Four BSC nurses were recruited for data collection. Training was given about tools and how to approach the participant by the investigator for data collectors and supervisors before the date of data collection.

### Data quality control

To ensure the quality of data, one-day training was provided for the data collectors, and supervisors in each referral hospital about techniques of data collection. Close supervision was given during the whole period of data collection, and each questionnaire was checked for completeness and consistency on a daily basis. The questionnaire was pre-tested on 5% [21] of the study subjects at Saint Paul’s Hospital Millennium Medical College (SPHMMC) to check the tools’ understandability and appropriateness.

### Data analysis and management

The collected data were checked for completeness and consistency, and then each questionnaire was coded and entered into Epi Data version 4.6.0 software and exported to SPSS version 25.0 for analysis. The Hosmer-Lemeshow test was checked to evaluate whether the assumption for binary logistic regression was fulfilled or to evaluate model fitness. The test result revealed a *P*-value of 0.6, which indicates that the assumption is fulfilled and the model is fitted with data. Multicollinearity was checked using the variance inflation factor (VIF), and the output indicated that there was no multicollinearity. A bivariable logistic regression was done, and variables with a *p*-value < 0.2 in a bivariable analysis were entered into a multivariable regression analysis to identify factors. A variable with a *P*-value of < 0.05 in the multivariable analysis was considered a measure of statistically significant determinants. The adjusted odds ratio with its 95% confidence interval was used to measure the strength of the association between variables. The results were described and presented in tables, graphs, and narrative descriptions [40].

## Result

### Socio demographic characteristics of the study populations

In this study, a total of 407 HIV positive women participated, giving a response rate of 99.5%. The mean age of participants was 42.5 years (SD = 9.4). One hundred fifty- three (37.6%) of the participants had attained the primary level of education. Regarding marital status, 141 (34.6%) of participants were married. Among the total respondents 176 (43.2%) of them were private employed. The Majority 262 (64.4%) of the study participants family monthly income had more than 2000 Ethiopian birr. (Table 1).

**Table 1:** Socio demographic characteristics of HIV positive women attending adult ART clinic in public hospitals, Addis Ababa, 2022

Variables	Frequency (*n*)	Percent (%)
**Age **(***n*** **=407)**		
≤45	261	64.1
>45	146	35.9
**Education (** ***n*** **=407)**		
No formal education	72	17.7
Primary level education	153	37.6
Secondary level education	133	32.7
Diploma and above	49	12
**Marital status (** ***n*** **=407)**		
Single	58	14.3
Married	141	34.6
Divorced	91	22.4
Widowed	117	28.7
**Occupation (=407)**		
Housewife	107	26.3
Government	72	17.7
Private	176	43.2
Unemployment	52	12.8
**Income (** ***n*** **=407)**		
≤1000birr	55	13.5
1001-2000birr	90	22.1
>2000birr	262	64.4

### Reproductive health and behavioral factors

The average age at first sexual intercourse of participants was 19.6 years (SD = 3.6). On the other hand, the majority 314 (77.1%) of participants gave birth. Respondents average number of children was 2.1 SD = 1.17). When it came to the utilization of contraceptives more than half 235 (57.7%) of participants had ever used contraceptive. Among those who had ever used contraceptives 125 (53.2%) of them had ever used contraceptives for more than five years duration. 374 (91.9%) and 242 (59.5%) of the study participants did not smoke and had multiple sexual partners. Regarding the perception of being susceptible to cervical cancer, 244 (60%) of the study participants were perceived as being susceptible to cervical cancer (Table 2).

**Table 2:** Reproductive health and behaviors of HIV positive women attending at ART clinic in public hospitals, Addis Ababa, 2022

Variables	Frequency (*n*)	Percent (%)
**Age at first sexual intercourse (** ***n*** **=407)**		
<18years	168	41.3
≥18years	239	58.7
**Ever give birth (** ***n*** **=407)**		
No	93	22.9
Yes	314	77.1
**Number of children (** ***n*** **=314)**		
<3	109	34.7
≥3	205	65.3
**Ever use contraceptive (** ***n*** **=407)**		
No	172	42.3
Yes	235	57.7
**Duration of contraceptive (** ***n*** **=235)**		
≤1000birr	110	46.8
>5years	125	53.2
**Have multiple sexual partners (** ***n*** **=407)**		
No	244	59.5
Yes	165	40.5
**Smoking (** ***n*** **=407)**		
No	375	91.9
Yes	33	8.1
**Known someone with cc (** ***n*** **=407)**		
No	268	65.8
Yes	139	34.2
**Susceptible to cc (** ***n*** **=407)**		
No	244	60
Yes	163	40

### Awareness and source of information

Among the total participants365 (89.7%) and 370 (90.9%) of the study participants had heard about cervical cancer and its screening respectively. For those participants who had information, health professionals 201 (55.1%) and 230 (62.3%) were the major sources of information about cervical cancer and its screening respectively. More than one response is possible (Table 3).

**Table 3:** Awareness and source of information of HIV positive women attending at adult ART clinic in public hospitals, Addis Ababa, 2022

**Variables**	**Frequency (** ***n*** **)**	**Percent (%)**
**Heard about cc (** ***n*** **=407)**		
No	42	10.3
Yes	365	89.7
**Source of information about cc (** ***n*** **=407)**		
Health professionals	201	55.1
Media	149	40.8
Friends and families	57	15.6
**Heard about ccs (** ***n*** **=407)**		
No	37	9.1
Yes	370	90.9
**Source of information about ccs (** ***n*** **=407**)		
Health professional	223	62.3
Media	128	36
Friends and families	56	15.2

### Knowledge and attitudes towards CC and CCS

A total of 407 HIV positive women participated the study, 220 (54.1%) and 233 (57.2%) had knowledge and a positive attitude towards cca. and ccs respectively (Table 4).

**Table 4:** knowledge and attitude of HIV positive women attending at adult ART clinic in public hospitals, Addis Ababa, 2022

**Variable**	**Frequency (n)**	**Percent (%)**
**Knowledge on cc and ccs (** ***n*** **=407)**		
Good knowledge	187	45.9
Poor Knowledge	220	54.1
**Attitude on cc and ccs(** ***n*** **=407)**		
Negative attitude	174	42.8
Positive attitude	233	57.2

### Clinical factors

Of the total participants, 245 (60.2%) and 226 (55.5%) have had 10 years or more. duration of HIV since diagnosed and ART follow up respectively (Table 5).

**Table 5:** clinical factors to uptake of cervical cancer screening among HIV positive women attending at public health hospitals, Addis Ababa, 2022

**Variable**	**Frequency (** ***n*** **)**	**Percent (%)**
**Duration of HIV since diagnosed (** ***n*** **=407)**		
<5years	61	15.0
5-9years	101	24.8
≥10years	245	60.2
**Duration of ART follow up (** ***n*** **=407)**		
<5years	63	15.5
5-9years	118	29
≥10years	226	55.5

### Uptake of cervical cancer screening

**Fig. 1:**
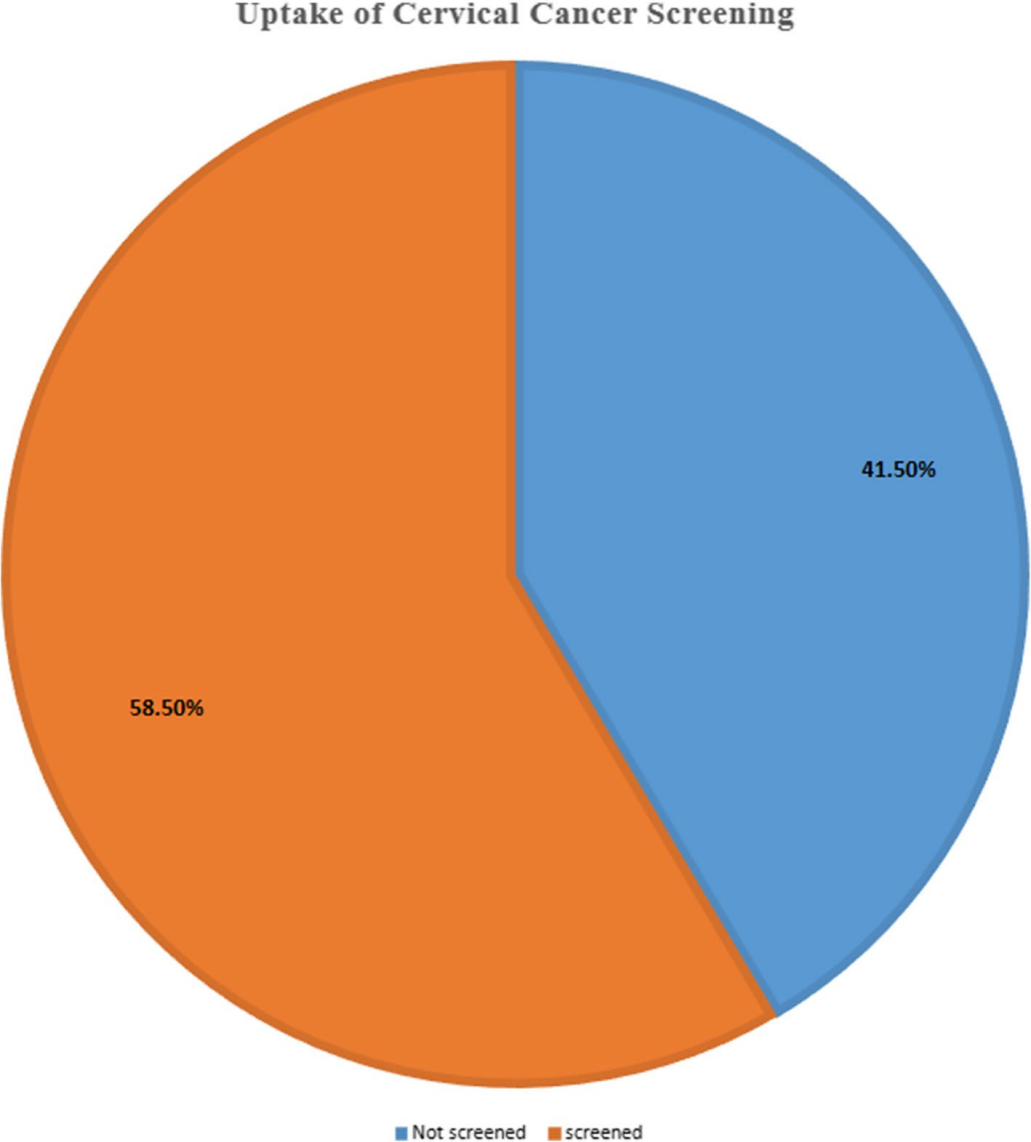
Uptake of cervical cancer screening among HIV positive women attending at ART clinic in public hospital, Addis Ababa, 2022

More than half 238 (58.5%), 95% CI (53.5–63.3) of participants have been screened at least once in the last 5 years since HIV was diagnosed (Fig. 1).

### Associated factors with uptake of cervical cancer screening

To identify the associated factors for uptake of cervical cancer screening, bi-variable and multivariable logistic regression analyses were carried out to see the association between independent variables and the outcome variable. Variables with a *p*-value < 0.2 on bivariable logistic regression were further run under multivariable logistic regression. Accordingly, variables including age group (> 45years), number of children (≥ 3children), perception of being susceptible to cervical cancer, knowledge, and good attitude towards cervical cancer were independent predictors of uptake of cervical cancer screening at a *p*-value of < 0.05 during multivariable analysis.

This study revealed that HIV positive women in the age group of > 45years old were 82% less likely to be screened than those in the age group of ≤ 45years old (AOR = 0.18-95% CI:0.053 − 0.644). Regarding the number of children, HIV positive women who had three or more children were 81% less likely to be screened than those with fewer than three children (AOR = 0.19, 95% CI: 036–0.979). HIV positive women who had a positive perception of being susceptible to cervical cancer were six times more likely to be screened than their counterparts (AOR = 6.39, 95% CI:1.797–22.744). Additionally, HIV positive women with knowledge about cervical cancer screening were 19 times more likely to be screened than their counterparts (AOR = 19.34, 95%CI: 7.87–47.75). Moreover, HIV positive women with a positive attitude towards cervical cancer screening were 8 times more likely to be screened than their counterparts (AOR = 8.06, 95%CI: 3.62–17.91) (Table 6).

**Table 6:** bivariable and multivariable analysis for selected variables on associated factors of uptake of cervical cancer screening among HIV positive women attending at ART clinic in public hospitals, Addis Ababa, 2022

**Variables**	**CC screening uptake**		**COR (95%CI)**	**AOR (95%CI)**	***P*** **-Value**
	No	Yes			
**Age**					
≤45years	75	186	1	1	
>45years	94	52	.22(.145-.344)	.18(.05-.64).008	
**Number of child**					
<3child	34	75	1	1	
≥3child	105	100	.43(.26-.70)	.19(.03-.97).047	
**Perception of susceptible to cc**					
No	134	110	1	1	
Yes	35	128	4.45(2.83-6.99)	6.39(1.79-22.74)	004
**Knowledge about ccs**					
No	131	56	1	1	
Yes	38	182	11.20(7.00-17.91)	19.34(7.87-47.50)	.001
**Attitude about ccs**					
Negative attitude	118	56	1	1	
positive attitude	51	182	7.52(4.82-11.72)	8.06(3.62-17.91)	.001

## Discussion

In this study, HIV positive women aged greater than 45 years, who had three or more children, had a positive perception of being susceptible to cervical cancer, and had knowledge and attitudes towards cervical cancer screening were significantly associated with the uptake of cervical cancer screening.

In this study, the uptake of HIV positive women was 58.5%,( 95% CI: (53.5–63.3).This studies finding is consistent with studies in Côte d’Ivoire at 59.7% [10] and in Northern Tanzania at (54%) [24]. However, it is higher than studies in Malawi 27.8% [41], Kenya 56% [42], Uganda (30.3%) [43], Nairobi (19%) [44], Hawassa (40.1%) [35], Bishoftu 25% [45], the Northwest, Ethiopia 23.5% [46], Addis Ababa (10.8%) [39] and Gondar, Ethiopia (10%) [36]. It can be explained that this might be due to the time difference, increased expansion of the service, awareness creation by using media promotions, characteristics of study participants, setting and integration of ART the clinic with the cervical cancer screening service, and improvement of ART the clinic staff’s counseling service to the patient about cervical cancer screening.

The current study showed that participants aged > 45 years were 82% less likely to be screened than those aged ≤ 45 years (AOR = 0.18, 95% CI: 0.05–0.64). This finding is in line with a study in Nandi County, Kenya, which reported that HIV-positive women aged between 30 and 39 years were more likely to be screened than women aged 50 years and above (34 (35.1%) vs. 9 (9.3%)) [47]. On the other hand, this finding contradicts studies in Côte d’Ivoire reported that HIV-positive women aged ≥ 45 years were 1.4 times more likely screened than aged < 45 years (AOR = 1.4, 95% CI: 1.1–1.8), in Nairobi showed that Women aged 35 years and above were two times more likely to have been screened than the younger ones (OR = 2.1; 1.1–3.9; *P* = 0.021) [10, 44]. Also, this finding contradicts studies conducted in Gondar by Mersha showed that HIV positive women aged between 30 and 39 years were nearly three times more likely to be screened than women aged < 29 years (AOR = 2.78, 95% CI : 1.71–7.29), and in Addis Ababa that HIV-positive women aged 40–49 years old were more likely to be screened than women age 18–29 years (36.1%) vs. (8%) [44, 48, 49]. This finding may be due the fact that to ART clinic staff counsel about cervical cancer screening and recommend that prioritized HIV positive women aged 15–49 years.

This study suggested that HIV-positive women who had at least three children were 81% less likely to be screened than those with fewer than three children (AOR = 0.19, 95% CI: 0.036-0.979). This finding is in line with a study in FinoteSelam that revealed that women who had a history of pregnancy of at least five were 80% less likely to be screened than nulliparous women (AOR = 0.2, 95% CI: 0.04–0.7) [5]. This finding contradicts a study in Dares Salaam showed that women who were grand multiparous were three times more likely to be screened than nulliparous women (AOR = 3.05, 95% CI:1.15–8.06) [50]. This variation might be related the fact that the majority of participants’ who underwent screening were younger and may have a small number of children.

It was found that HIV-positive women who had a positive perception of being susceptible to cervical cancer were six times more likely to be screened than their counterparts (AOR = 6.39_95% CI:1.79–22.74). This finding contradicts a study in Uganda, which reported that HIV-positive women who have a low perception of susceptibility to CC were 1.5 times more likely to be screened than their counterparts [43]. This finding is supported by studies by Mersha and Mekonen that reported that HIV-positive women who had a positive perception of susceptibility to cervical cancer were 2.85 times (AOR = 2.85, 95% CI:1.89–6.16) and 3.26 times (AOR = 3.26, 95% CI : 2.26–4.26) more likely to be screened than their counterparts respectively [32, 48]. Furthermore, a study by Woldetsadik revealed that women who perceived the chance of getting cervical cancer as likely were five times more likely to be screened than women who perceived the chance of getting cervical cancer as unlikely(AOR = 5.36, 95%CI: 2.12–13.55) [49]. It is obvious that women who have a positive perception of being susceptible to the disease may undergo screening.

HIV-positive women who had knowledge about cervical cancer screening were 19 times more likely to be screened than their counterparts (AOR = 19.34, 95% CI: 7.87–47.75). This finding is consistent with a study in Malawi that reported that HIV-positive women who had adequate knowledge were 0.1 times more likely screened than those who did have adequate knowledge [41]. Similarly, studies by Mersha and Mekonen revealed that HIV positive women who had good knowledge of cervical cancer and screening were 3.02 times and 3.26 times more likely to be screened than their counterparts respectively (32, 61). This contradicts a study in Adigrat that reported that those not knowing about CCS uptake were 1.8 times more likely to be screened than their counterparts (AOR = 1.8, 95% CI: 1.15, 2.69) [37]. It is also clear that knowledgeable women may undergo screening to prevent the disease and receive early treatment if they have it.

Moreover, HIV-positive women with a positive attitude towards cervical cancer screening were eight times more likely to be screened than their counterparts (AOR = 8.06, 95% CI: 3.62–17.91). Similarly, this study is supported by a study in Malawi, and Hawassa reported that HIV positive women who had a positive attitude toward screening were more likely to be screened than those who had a negative attitude (AOR = 1.36, 95% CI: 1.04–1.76) and (AOR = 3.7, 95% CI: 1.8–7.5) respectively [39, 44]. a study in FinoteSelam showed that women who had a positive attitude about CCS were 9 times more likely screened than their counterparts (AOR = 9, 95% CI: 1.5–18) [5]. This can be explained women who have positive attitude may undergo screening.

### Implication for practice

in this study findings, as the factor for uptake of cervical cancer screening service were knowledge and attitude of women towards cervical cancer and its screening, this calls the healthcare authority should create awareness through information, education and communication (IEC), and behavioral change communication (BCC) through health information communication on usage of cervical cancer screening. It is hoped that this.

will also improve the knowledge and attitude of women. Policymakers like the Ethiopian Ministry of Health (MoH) should ensure that continuing education programs on the use of cervical cancer screening for the prevention of invasive cancer.

#### Strengths and limitations of the study

The authors advise readers to exercise caution when interpreting any of the study’s findings because recollection and social desirability biases may have been present. However, this multi-center study offers important details concerning women’s use of cervical cancer screening services and its contributing factors.

## Conclusion

Among women who were HIV positive in this study, there was a low rate of cervical cancer screening uptake. A perception of being susceptible to cervical cancer, good knowledge of cervical cancer screening, and a positive attitude towards cervical cancer screening were all significantly associated with the uptake of cervical cancer screening services among HIV positive women in the age group of > 45 years who had > = 3 children. When creating and implementing plans to increase the use of CCA screening services in Addis Ababa, results should be taken into account.

## Data Availability

The datasets used and/or analyzed during the current study are available from the corresponding author upon reasonable request.

## Abbreviations

AOR: Adjusted Odd Ratio
ART: Antiretroviral therapy
CC: Cervical Cancer
CCS: Cervical Cancer Screening
CIN: Cervical Intraepithelial Neoplasm
COR: Crude Odd Ratio
EDHS: Ethiopian Demographic Health Survey
ETB: Ethiopian Birr
HIV: Human Immune Deficiency Virus
hrHPV: Highly Persistent Human Papilloma Virus
HPV: Human Papilloma Virus
LMIC: Low and Middle-Income Countries
SPSS: Statistical Package for Social Science
VIA: Visual Inspection with Acetic Acid
VILI: Visual Inspection with Lugol's Iodine Solution
